# Assessment of biorisk management systems in high containment laboratories, 18 countries in Europe, 2016 and 2017

**DOI:** 10.2807/1560-7917.ES.2020.25.36.2000089

**Published:** 2020-09-10

**Authors:** Sandra Appelt, Daniela Jacob, Anna-Maria Rohleder, Andreas Bråve, Åsa Szekely Björndal, Antonino Di Caro, Roland Grunow, on behalf of the Joint Action EMERGE laboratory network

**Affiliations:** 1 Centre for Biological Threats and Special Pathogens (ZBS2), Robert Koch Institute, Berlin, Germany; 2 Office of Department of Microbiology, Public Health Agency of Sweden, Solna, Sweden; 3 Lazzaro Spallanzani National Institute for Infectious Diseases, Rome, Italy; 4 The EMERGE partners are acknowledged at the end of the article

**Keywords:** biosafety, biosecurity, bioterrorism, laboratory, public health terms

## Abstract

Europe-wide activities to improve biosafety and biosecurity performed within the frameworks of the European Union (EU)-funded Joint Actions EMERGE and QUANDHIP led to the development of an Integrated European Checklist for Laboratory Biorisk Management (ECL).

To better understand different approaches shaping biorisk management (BRM) systems on an operational level in high containment laboratories, the ECL was used to map the implementation of BRM in 32 high containment laboratories in 18 countries in Europe. The results suggest that the BRM elements referring to standard microbiological working practices and the handling of infectious material were fulfilled particularly well. The elements safety exercises involving internal and external emergency responders, and appropriate decommissioning plans were not fulfilled particularly well. BRM in Biosafety Level (BSL) 4 laboratories handling Risk Group (RG) 4 viruses appear to vary among each other less than BSL3 laboratories handling RG 3 bacteria. It is important to agree on comparable regulations in Europe as high containment laboratories are indispensable for a safe, quick and effective response to public health threats. As high containment laboratories may also present a public health risk it is crucial to have robust BRM on organisational and operational levels.

## Background

Emerging and re-emerging pathogens are of public health significance, especially those agents classified as Risk Group (RG) 3 and 4 which have the potential to cause public health emergencies, e.g. *Bacillus anthracis, Yersinia pestis*, filoviruses or arenaviruses. These agents pose a high risk for individuals and communities, causing severe to lethal disease in humans and animals depending on the infection route. The handling of such agents is usually restricted to high containment facilities to minimise a possible public health risk. RG3 agents are handled in Biosafety Level (BSL) 3 and RG4 agents in BSL4 laboratories [1].

For the work in high containment laboratories, biorisk management (BRM) systems, including adequate biosafety and biosecurity measurements, should be established to prevent the release of or exposure to infectious material [1,2]. Although, it was assumed that adequate BRM systems would help to achieve an appropriate level of biosafety by determining best laboratory biosafety practices and by reinforcing biosecurity systems [1,3-8], high containment facilities have experienced safety and security breaches [9-13]. Specific requirements for high containment laboratories were determined, and corresponding guidelines at national and international levels were developed, released and implemented. A manual applied widely at the international level is the World Health Organization (WHO) Laboratory biosafety manual, which underscores the need to have appropriate containments [1]. A list of guidance documents and biosafety associations is provided in Supplementary Table S1.

In the framework of the Joint Action QUANDHIP (Quality Assurance Exercises and Networking on the Detection of Highly Infectious Pathogens) [14] funded by the European Union (EU) Health Programme 2014–2020, an additional tool was developed: the Integrated European Checklist for Laboratory Biorisk Management (ECL) [15]. This checklist, which is freely available (www.emerge.rki.eu/Emerge/EN/Content/Quandhip/quandhip_node.html) [14], allows high containment laboratories to have an external or internal evaluation of biocontainment requirements by responding to ECL items (checkboxes) dedicated to 14 BRM elements (Table 1). The ECL was agreed between 29 BSL3 laboratories and six BSL4 laboratories in Europe and contributes to establishing commonly and mutually accepted BRM recommendations. This facilitates the setting-up of new laboratories and the self-evaluation of existing laboratories based on internationally-accepted BRM practices. High containment laboratories are indispensable for a safe and effective response to public health threats. However, the ECL does not replace national regulations or guidelines. We used the tool to perform an assessment of the operational level of BRM systems in European laboratories to determine and discuss potential gaps that could lead to safety and security breaches.

**Table 1:** Number of participating laboratories in full compliance with the indicated elements of the ECL, 18 countries in Europe, assessed 2016­–2017

ECL chapter	BRM element of the ECL		Number of ECL items (i.e. checkpoints) referring to a BRM element			Number of high containment laboratories in full compliance with a BRM element^a^		
			BSL3	BSL4 suited^b^	BSL4 cabinet lines^c^	BSL3 (n = 25)	BSL4 suited^b^ (n = 5)	BSL4 cabinet lines^c^ (n = 2)
**2**	Laboratory design and infrastructure		12	17	15	12	3	1
**3**	BSCs and BSC lines		4	3	7	11	2	2
**4**	Containment barrier – heating, ventilation and air conditioning		17	16	16	9	4	0
**6**	Laboratory integrity of facilities including surface finishes and case work		8	9	6	15	3	1
**7**	Containment perimeter		6	6	7	22	5	2
**8**	Personnel and chemical shower plant operation and laboratory services		8	11	12	12	4	2
**9**	Emergency provision, plans and responses		25	30	29	7	3	1
**10**	Planned preventative maintenance, calibration and certification records		18	26	23	8	3	1
**11**	Commissioning and decommissioning		7	7	7	10	3	1
**14**	Personal protective equipment		9^d^ or 8^e^	8	7	12	0	1
**15**	Personnel recruitment, competence and training		23	23	23	13	5	2
**16**	Operational procedures and special practices	Standard microbiological and work practices	12	12	12	18	5	2
		Handling infectious material	6	6	5	22	5	2
		Handling of sharps	2	2	2	16	4	1
		Compressed gas cylinders^f^	10	10	10	15	4	2
**17**	Biosecurity	Physical security measures in place	7	7	7	16	5	1
		Personnel-suitability and reliability	9	9	9	10	3	1
		Pathogen accountability	12	12	12	14	4	1
**18**	Summary of required documentations		15	15	15	14	4	1

BRM: biorisk management; BSC: biological safety cabinet; BSL: biosafety level; ECL: Integrated European Checklist for Laboratory Biorisk Management; NA: not applicable.

^a^ Results based on 25 BSL3 laboratories handling RG3 bacteria and seven BSL4 laboratories handling RG4 viruses.

^b^ BSL4 suited: a suit laboratory where air-supplied, pressurised protective suits are used in the biological environment as personal protective equipment by laboratory personnel.

^c^ BSL4 cabinet lines: the laboratory environment consists of cabinet lines.

^d^ BSL3 designed using BSC Class II cabinets.

^e^ BSL3 designed using BSC Class III cabinets.

^f^ The BRM element was not applicable for 11 containment laboratories.

## Participation and assessment process

Participating study centres were European BSL3 and BSL4 facilities officially approved by national authorities for the diagnostics of RG3 bacteria and/or RG4 viruses according to the national rules that were nominated by the competent authorities of their countries to participate in the two Joint Actions mentioned above. They therefore took part in the application of the ECL as part of the project. To obtain information about their procedures for BRM in containments, the ECL [15] (www.emerge.rki.eu/Emerge/EN/Content/Quandhip/quandhip_node.html) was completed and signed by their biosafety officers. The participation and application of the ECL was agreed in a Consortium Agreement in the framework of the Joint Action EMERGE.

Because of security issues and data protection, the participating BSL3 and BSL4 laboratories were anonymised by individual identifiers.

The 14 chapters of the ECL, one chapter per BRM element, were used to assess the implementation of BRM elements in high containment laboratories. The most complex elements, with 30 items (i.e. checkpoints) each, are dedicated to operational procedures, special practices and emergency provision, plans and responses. The less complex elements, with seven items each, cover containment perimeter requirements, commissioning and decommissioning. The filled-in checklists completed by each of the 32 laboratories were analysed for completeness by us. Each item of each ECL chapter was weighted as one task [16,17]. The data were collected between 2016 and 2017. The results gathered for each of the high containment laboratories were compared and analysed for BSL3 (bacteria) and BSL4 (viruses) laboratories. To check if the evaluation might be biased by ECL chapter complexity, i.e. some chapters consist of more items than others, the item difficulty of each BRM element was statistically calculated. The item difficulty of each ECL element was defined as the sum of reached points by all participants divided by the sum of reachable points [17-19]. Based on this, one can conclude how difficult it might be for a randomly selected facility to fulfil a specific BRM element. The item difficulty ranged between 0.83 and 1.00 (Supplementary Table S2). Consequently, the item difficulty of a BRM element was not influenced by the chapter complexity.

## Limitations

Collected data were obtained from self-assessments, a direct confirmation of transmitted information was not performed and authors rely on the accuracy of provided information. In the future, on-site evaluation by invited external experts would further guarantee an objective assessment. For security reasons, results related to possible security issues and details about participating laboratories in European countries were excluded.

## Fulfilment of biorisk management elements

A total of 32 BSL3 and BSL4 facilities from 18 European countries were assessed, comprising 25 BSL3 laboratories (bacteria), five BSL4 suited laboratories (viruses) and two BSL4 holding cabinet lines. The 14 investigated BRM elements were completely fulfilled by at least 11 of 32 and at most by 29 of 32 (Table 1) containment laboratories. Fulfilled by 11 facilities only, was the element about emergency provision, including plans and responses, and the elements about containment perimeters. Also, one of the most challenging BRM elements identified was the handling of sharps, including the implementation of policies for a safe handling and usage of needle-locking or disposable syringes. The easiest BRM elements to fulfil comprised standard microbiological working practices and the handling of infectious material.

Basic infrastructure and design conformity is given in nearly all examined high containment laboratories. They are separated from public areas; containments are labelled and the access is restricted and controlled (30/32 laboratories; Table 1). We found that especially new facilities are separated from external building boundaries (6/25 BSL3 laboratories; 1/7 BSL4 laboratories). Standard microbiological working practices were implemented by almost all participating laboratories (Table 1 and Table 2): infectious material is stored in safe containers, decontamination and inactivation procedures are adapted to the handled agents, usage of glassware is avoided and physical security measures are in place to protect workers and laboratory environments from being exposed to agents. Tracking systems inform about the procession and inventory of biological material and records are kept (23/25 BSL3 laboratories; 6/7 BSL4 laboratories). For waste management, double-door autoclaves are available (23/25 BSL3 laboratories; 7/7 BSL4 laboratories) beneath alternative gas and chemical treatments (32/32 laboratories). Results show that physical integrity tests of buildings are rarely performed (5/32 laboratories). The same applies to emergency trainings involving external first-line responders (5/25 BSL3 laboratories; 5/7 BSL4 laboratories), including the transfer of injured persons to medical centres (6/25 BSL3 laboratories; 5/7 BSL4 laboratories) or responses to natural disasters (5/25 BSL3 laboratories; 5/7 BSL4 laboratories). Commissioning measurements are available, whereas decommissioning measures (13/25 BSL3 laboratories; 3/7 BSL4 laboratories), including relevant national regulations or agreements (9/25 BSL3 laboratories; 1/7 BSL4 laboratories), do not seem to be in place (Table 3). Regarding biosecurity, it turned out that in some laboratories, photo identification of employees is not performed (9/25 BSL3 laboratories; 3/7 BSL4 laboratories) and security checks of staff members are not yet carried out (3/25 BSL3 laboratories; 0/7 BSL4 laboratories) which might cause a major security concern.

**Table 2:** Level of fulfilment^a^ of BRM elements, 32 high containment laboratories in Europe, 2016–2017

ECL chapter	BRM element of the ECL		Laboratory containment level																															
			BSL3 (n = 25)																									BSL4 suited^b^ (n = 5)					BSL4 cabinet line^c^ (n = 2)	
Laboratory number			1	2	3	4	5	6	7	8	9	10	11	12	13	14	15	16	17	18	19	20	21	22	23	24	25	26	27	28	29	30	31	32
**2**	Laboratory design and infrastructure		1.00	0.94	1.00	0.94	0.94	1.00	1.00	0.94	1.00	1.00	1.00	1.00	0.94	0.88	1.00	0.94	0.94	1.00	0.88	1.00	0.94	0.88	0.94	0.75	0.88	1.00	0.88	1.00	1.00	0.88	0.87	1.00
**3**	BSCs and BSC lines		1.00	0.96	1.00	1.00	0.96	1.00	1.00	0.96	1.00	0.96	0.96	1.00	0.96	0.96	1.00	0.96	1.00	1.00	1.00	0.96	0.96	0.96	0.96	0.96	0.87	1.00	0.95	0.95	0.95	0.95	1.00	1.00
**4**	Containment barrier – heating, ventilation and air conditioning		1.00	1.00	1.00	1.00	0.95	0.84	0.95	0.84	1.00	1.00	1.00	0.95	1.00	0.79	0.89	0.89	0.95	0.89	0.89	1.00	0.84	0.58	0.89	0.58	0.68	1.00	1.00	1.00	1.00	0.95	1.00	0.85
**6**	Laboratory integrity of facilities including surface finishes and case work		1.00	0.89	1.00	1.00	1.00	0.89	1.00	1.00	1.00	1.00	1.00	1.00	1.00	0.89	1.00	0.89	1.00	0.78	0.89	0.89	1.00	0.67	0.89	1.00	0.78	1.00	0.89	0.89	1.00	1.00	1.00	0.83
**7**	Containment perimeter		1.00	1.00	1.00	0.75	1.00	1.00	1.00	1.00	1.00	1.00	1.00	1.00	1.00	1.00	1.00	1.00	1.00	1.00	0.88	1.00	1.00	0.63	1.00	1.00	1.00	1.00	1.00	1.00	1.00	1.00	1.00	1.00
**8**	Personnel and chemical shower plant operation and laboratory services		1.00	1.00	1.00	1.00	1.00	1.00	0.77	0.85	1.00	1.00	1.00	0.77	0.92	0.92	1.00	1.00	0.92	0.85	0.92	0.85	0.92	0.69	1.00	0.69	0.77	1.00	1.00	1.00	1.00	0.77	0.92	1.00
**9**	Emergency provision, plans and responses		1.00	1.00	0.96	1.00	1.00	1.00	0.96	0.91	0.83	0.78	0.88	0.79	0.96	0.88	1.00	0.96	1.00	0.83	0.88	0.88	1.00	0.88	0.83	0.79	0.61	1.00	0.95	1.00	1.00	0.75	1.00	0.82
**10**	Planned preventative maintenance, calibration and certification records		0.96	0.96	0.96	1.00	1.00	1.00	0.87	0.96	1.00	0.96	1.00	0.83	1.00	0.91	1.00	0.96	1.00	0.91	0.96	0.91	0.83	0.78	0.96	0.96	0.74	1.00	0.92	1.00	0.92	1.00	0.96	1.00
**11**	Commissioning and decommissioning		0.71	1.00	1.00	1.00	1.00	0.71	1.00	0.71	0.86	0.86	1.00	0.71	1.00	1.00	0.86	1.00	0.86	0.71	0.71	0.71	0.86	1.00	0.86	0.86	0.00	1.00	1.00	0.71	0.86	1.00	1.00	0.86
**14**	Personal protective equipment		1.00	0.89	1.00	0.78	1.00	0.78	0.78	1.00	0.89	0.89	1.00	1.00	0.89	0.78	0.89	0.89	1.00	1.00	1.00	0.89	1.00	1.00	1.00	0.89	0.89	0.88	0.89	0.75	0.88	0.38	1.00	0.83
**15**	Personnel recruitment, competence and training		1.00	1.00	1.00	1.00	0.96	1.00	0.96	1.00	1.00	0.87	0.91	1.00	1.00	0.91	1.00	0.96	1.00	1.00	0.87	0.96	0.65	1.00	0.87	0.96	0.91	1.00	1.00	1.00	1.00	1.00	1.00	1.00
**16**	Operational procedures and special practices	Standard microbiological working practices	1.00	1.00	1.00	1.00	1.00	1.00	0.92	1.00	1.00	1.00	0.83	0.92	1.00	1.00	1.00	0.83	1.00	1.00	0.83	1.00	1.00	1.00	0.83	0.92	1.00	1.00	1.00	1.00	1.00	1.00	1.00	1.00
		Handling infectious material	1.00	1.00	1.00	1.00	1.00	1.00	1.00	0.83	1.00	1.00	1.00	1.00	1.00	1.00	1.00	1.00	1.00	1.00	1.00	0.83	0.83	1.00	1.00	1.00	1.00	1.00	1.00	1.00	1.00	1.00	1.00	1.00
		Handling of sharps	1.00	1.00	1.00	1.00	1.00	1.00	1.00	1.00	0.50	0.50	1.00	1.00	0.50	1.00	0.00	1.00	1.00	0.50	1.00	0.50	0.50	1.00	0.50	0.50	1.00	1.00	1.00	1.00	0.00	1.00	1.00	0.50
		Compressed gas cylinders	1.00	1.00	1.00	1.00	1.00	1.00	NA	NA	1.00	1.00	1.00	NA	NA	NA	1.00	1.00	NA	1.00	NA	1.00	NA	NA	NA	1.00	1.00	1.00	1.00	1.00	1.00	NA	1.00	1.00
**17**	Biosecurity	Physical security measures in place	1.00	1.00	1.00	1.00	1.00	1.00	0.86	1.00	0.86	1.00	0.86	0.86	0.71	1.00	1.00	0.71	1.00	1.00	1.00	1.00	1.00	1.00	0.86	0.86	0.57	1.00	1.00	1.00	1.00	1.00	1.00	0.86
		Personnel-suitability and reliability	0.78	1.00	1.00	1.00	1.00	1.00	1.00	1.00	0.67	0.89	0.78	0.89	0.89	0.89	0.89	1.00	0.89	1.00	0.89	0.89	0.78	1.00	0.67	0.89	0.56	1.00	1.00	1.00	0.89	0.78	1.00	0.67
		Pathogen accountability	1.00	1.00	1.00	1.00	0.92	1.00	1.00	1.00	0.83	1.00	0.75	1.00	0.83	0.75	1.00	1.00	1.00	1.00	0.75	0.92	1.00	0.92	0.92	0.83	0.50	1.00	1.00	1.00	1.00	0.92	1.00	0.83
**18**	Summary of required documentations		1.00	1.00	NI	1.00	0.93	0.93	1.00	1.00	0.93	0.67	0.93	1.00	1.00	1.00	1.00	0.93	1.00	0.87	0.87	1.00	1.00	1.00	0.93	1.00	0.93	1.00	1.00	0.93	1.00	0.93	1.00	0.93

BRM: biorisk management; BSC: biological safety cabinet; BSL: biosafety level; ECL: Integrated European Checklist for Laboratory Biorisk Management; NA: not applicable; NI: no information.

^a^ The level of fulfilment a BRM element was calculated as sum of fulfilled tasks divided by the total number of tasks.

^b^ BSL4 suited: is a suit laboratory where air-supplied, pressurised protective suits are used in the biological environment as personal protective equipment by laboratory personnel.

^c^ BSL4 cabinet lines: the laboratory environment consists of cabinet lines.

The colour code reflects the level of fulfilment of a BRM element: red (0–39%), orange (40–69%) and green (70–100%, with lighter shades suggesting lower levels of fulfilment).

**Table 3:** ECL items (i.e. checkpoints) of BRM elements listed in the ECL not available in some of the 32 assessed high containment laboratories in Europe, 2016–2017

Items of BRM listed in the ECL	Number of laboratories NOT in compliance with ECL items	
	BSL3 (n = 25)	BSL4 (n = 7^a^)
Implementation of waste management and decontamination strategies	3	0
Personnel security check ups	3	0
Necropsy tables equipped with HEPA-filtered downdraughts	NA	4
Tests of power interruption supplies (e.g. standby power, UPS systems)	4	0
Overrides of emergency exit doors, needed to open doors without other devices	4	1
Staff members criminal background checks	5	1
For maintaining a positive pressurisation in containment laboratories, supply air systems need to be interlocked with exhaust air systems	6	0
Laboratories are supplied with HEPA-filtered outside air	6	0
Exhausted air of the laboratory environment is double HEPA-filtered before release	6	0
Protection of water supplies against contamination by installing backflows, isolation valves and drainage traps	6.5	0
Annual emergency exercises	8	0
Tests for interlocking of doors and water shower systems	8	0
Interlocking test strategies for Class II Type B2^b^ BSCs to turn off internal supply fans whenever exhaust fans fail^c^	5	4
Decommissioning measures or national regulations or agreements on the decommissioning of high containments	14	5
Simulation of injured person transfer to medical centres during emergency trainings	19	2
Involvement of emergency responder into emergency trainings	20	2
Training of responses to natural disasters	20	2
Validation of applied procedures for liquid effluent systems	19	6
Off-site emergency team protective overall suits	22	5
To prevent leaking of fluids, perimeter frames for open-seamed bench tops are needed as well as catches in drawers to prevent them from dropping down	22	5

BRM: biorisk management; BSC: biological safety cabinet; BSL: biosafety level; ECL: Integrated European Checklist for Laboratory Biorisk Management; HEPA: High Efficiency Particulate Air; NA: not applicable.

^a^ Includes five BSL4 suited (high containment laboratories applying suits as personal protective equipment insight the containment) and two BSL4 cabinet lines (personal protection is given by appropriate biosafety cabinets) .

^b^ In this type of BSC, none of the air (inflow, downflow) is recycled.

^c^ Not applicable for the two BSL4 cabinet lines.

The list is arranged from least to most non-compliant laboratories: green (0–4 non-compliant laboratories), orange (5–10 non-compliant) and red (11–22 non-compliant).

The findings outlined by the examples in Table 3 could show that BRM elements scored differently for BSL3 than for BSL4. No entire BRM element was fulfilled by all BSL3 laboratories, whereas four entire BRM elements were fulfilled by all BSL4 laboratories. However, the results might be biased because of the low number of investigated BSL4 laboratories.

## Differences among laboratories of one country and in comparison to other countries

We also looked at: (i) differences between high containment laboratories of the same type in the same country and (ii) differences between high containment laboratories of the same type in different European countries. According to our results, which show that BRM elements are fulfilled differently in all laboratories (Table 2), and that BSL3 laboratories (bacteria) from the same country considerably differ in their implementation of BRM elements (data not shown), it can be assumed that the variations in BSL3 laboratories in one country are comparably diverse to variations observed in laboratories of different countries.

In contrast, for BSL4 laboratories (viruses), the rather low sample size makes it difficult to compare within and between countries. It preliminarily seems that the BRM elements in place in international BSL4 laboratories for the containment of viruses vary little among each other at the European level, although further information is still required.

## Discussion

Our main focus was to assess BRM system implementation in high containment laboratories in Europe, to identify critical points and to present a comparison of BRM practices. This could contribute to establishing a consensus about comparable regulations in Europe, as high containment laboratories are indispensable for a safe, quick and effective response to public health threats. The potential public health risk of these laboratories is minimised if best practice of safety and security regulations is implemented.

In high containment laboratories, for the protection of workers, the community and the environment against exposure to highly infectious material, BRM plans, biosafety protocols and laboratory biosecurity measurements are essential [1,6,11,20]. However, putting international and European guidelines into practice requires adequate resources which should be considered when planning these high containment laboratories.

Our assessment of BRM systems in Europe showed that for BSL3 laboratories, differences between and within countries are present (Table 2). No laboratory was alike, possibly because comprehensive national laws and regulations are missing. We found that decommissioning measures for high containments are not regulated or controlled by federal or independent authorities for 25 of 32 laboratories in European countries. Also, preliminary data indicate that BSL4 laboratories seem to have been constructed on the basis of an international consent and exchange of experiences, but further investigations are still needed for confirmation.

Validation of liquid effluent treatment systems should be essential and never be missed in case decontamination is needed for water from showers or autoclave chambers. We also found that off-site emergency team overall suits are not available in the surrounding area of almost all investigated laboratories which might hamper rescue operations. Also, it seems that perimeter frames for open-seamed bench tops are needed and that catches, which prevent drawers from dropping down, are missing in 27 of 32 laboratories. Moreover, based on our assessment, improvement is also clearly needed for safety exercises with internal and external emergency responders, as well as for exercising responses to natural disasters.

We could also show that there are variations among the 32 facilities: some are technologically more advanced than others. This might be due to varying resources, the year of construction and amended regulations. In contrast, it appears that there are only a few variations between BSL4 laboratories. Checking the suitability of persons having access to facilities may need to be made mandatory. In the future, it would be desirable to include more BSL4 facilities in the evaluation of BRM systems to get a more comprehensive and conclusive overview. However, we would still expect to find little variation as BSL4 laboratories are generally under strict national control because of public health risks and elevated public interest. The compliance of BRM plans, biosafety protocols and laboratory biosecurity measurements in BSL4 environments is essential as there is no effective treatment against RG4 agents.

The BRM element that scored best in 29 of 32 laboratories was related to operational procedures and microbiological practices as well as to the handling of infectious material, strictly controlled by staff working within BSL3 and 4 laboratories. Both topics are important pillars of high-quality BRM systems preventing workers from being exposed to life-threatening pathogens [2]. Since animal tests are rarely performed in the investigated laboratories, the absence of procedures to ensure the safe handling of sharps is less surprising.

## Conclusions

Further improvements and continuous quality assurance via self-assessment, and ideally also through external review, are needed. Our findings could be used to implement such improvements, including the enhancement or updating of BRM systems, as well as the setting-up of new containments with robust BRM systems. Further strengthening of cooperation by developing trustful and transparent relations, by pro-actively accumulating knowledge, by sample sharing and by exchanging best practices is necessary to achieve a harmonisation of standards at the European level. Also, the implementation of the International Health Regulations will continuously support the strengthening of BRM in high containment laboratories.

## Supplementary Data

55900 Supplementary Material
