# Supplementary material for: Assessment of biorisk management systems in high containment laboratories, 18 countries in Europe, 2016 and 2017
Source: Euro Surveill. 2020 Sep 10;25(36):2000089. doi: 10.2807/1560-7917.ES.2020.25.36.2000089 (PMC7502897; doi:10.2807/1560-7917.ES.2020.25.36.2000089)
Supplement: Supplementary Material [file 20-00089_GRUNOW_Supplement.pdf]

Supplementary material

This supplementary material is hosted by *Eurosurveillance* as supporting information alongside the article 'Assessment of biorisk management systems in high containment laboratories, 18 countries in Europe, 2016 and 2017' on behalf of the authors, who remain responsible for the accuracy and appropriateness of the content. The same standards for ethics, copyright, attributions and permissions as for the article apply. *Supplements are not edited by Eurosurveillance and the journal is not responsible for the maintenance of any links or email addresses provided therein*

Supplementary Table S1: Exemplary listing of guidance documents and associations helping with the implementation of BRM systems in high-level containments. (Please, see last pages of the manuscript.)

Guidance documents	Publisher	Web-link
Laboratory biosafety manual*	WHO: World Health Organization	https://apps.who.int/iris/handle/10665/42981
Responsible life sciences research for global health security	WHO	https://apps.who.int/iris/handle/10665/70507
Biosafety in microbiological and biomedical laboratories	US Deptartment of Health and Human Services CDC: Centers for Disease Control and Prevention	https://www.cdc.gov/labs/pdf/CDC-BiosafetyMicrobiologicalBiomedicalLaboratories-2009-P.PDF
Guidelines for safe work practices in human and animal medical diagnostic laboratories.	CDC: Centers for Disease Control and Prevention	https://www.cdc.gov/mmwr/preview/mmwrhtml/su6101a1.htm
Biosafety buyer's guide	ABSA: Association for Biosafety and Biosecurity	https://absa.org/news/2017-12-biosafety-buyers-guide/
Directive 2000/54/EC	European parliament and the council	https://eur-lex.europa.eu/legal-content/EN/ALL/?uri=CELEX%3A32000L0054
CEN Workshop Agreement, CWA 15793, laboratory biorisk management	European committee for standardization	https://absa.org/wp-content/uploads/2017/01/CWA15793_Feb2008.pdf
CEN Workshop Agreement, CWA 16393, laboratory biorisk management – guidelines for the implementation of CWA 15793	European committee for standardization	https://www.cdc.gov.tw/Uploads/files/201504/d0feebf2-a92c-46e1-914a-b9d1435bc52f.pdf
Technical Rules for Biological Agents, TRBA 100, Protective measures for activities involving biological agents in laboratories	Committee for Biological Agents	https://www.baua.de/EN/Service/Legislative-texts-and-technical-rules/Rules/TRBA/pdf/TRBA-100.pdf?__blob=publicationFile&v=2
ISO 35001:2019	Biorisk management for laboratories and other related organisations	https://www.iso.org/standard/71293.html
**Association**		
EBSA: European Biosafety Association	European Biosafety Association	https://ebsaweb.eu/
IFBA: International Federation and Biosafety Associations	International Federation and Biosafety Associations	https://internationalbiosafety.org/
International Health Regulation tools	WHO	https://www.who.int/ihr/en/
* the fourth version of the Laboratory biosafety manual will be available soon		

Supplementary Table S2: Shown are the BRM elements covered by the ECL, the number of tasks per element dependent on the containment level as well as the item difficulties of each element. The item difficulty of each BRM element was calculated by means of all participants (n = 32). The item difficulty expresses how difficult it is to fulfill a given task by means across all participants. The easiest tasks to fulfill are highlighted in yellow and the most difficult tasks in blue.

ECL Chapter	BRM elements (ECL element)	**Number of tasks** (e.g checkpoints) **referring to one BRM element dependent on containment levels**			Item difficulty of an BRM element
		**BSL-3**	**BSL-4 Suited**	**BSL-4 Lines**	
2	Laboratory design and infrastructure	12	17	15	0.94
3	Biological safety cabinets (BSCs) and BSC lines	4	3	7	0.88
4	Containment barrier – heating, ventilation and air conditioning	17	16	16	0.88
6	Laboratory integrity of facilities including surface finishes and case work	8	9	6	0.86
7	Containment perimeter	6	6	7	0.95
8	Personnel and chemical shower plant operation and laboratory services	8	11	12	0.89
9	Emergency provision, plans and responses	25	30	29	0.87
10	Planned preventative maintenance, calibration and certification records	18	26	23	0.93
11	Commissioning and decommissioning	7	7	7	0.91
14	Personal protective equipment	9°-8°°	8	7	0.94
15	Personnel recruitment, competence, and training	23	23	23	0.96
16	Operational procedures and special practices				
16	- Standard microbiological and work practices	12	12	12	0.97
16	- Handling infectious material	6	6	5	0.98
16	- Handling of sharps	2	2	2	0.83
16	- Compressed gas cylinders#	10	10	10	1.00#
17	Biosecurity				
17	- Physical security measures in place	7	7	7	0.94
17	- Personnel-suitability and reliability	9	9	9	0.87
17	- Pathogen accountability	12	12	12	0.90
18	Summary of required documentations	15	15	15	0.93

°BSL 3 designed using BSC Class II; °°BSL 3 designed using BSC Class III # for a total of 12 laboratories the point was not applicable, therefore the element cannot be rated as the element fulfilled the best
